# Welsh Firms and the Insurance Act

**Published:** 1912-09-21

**Authors:** 


### Welsh Firms and the Insurance Act.

The effects of the National Insurance Act from quite different points of view were discussed, writes a correspondent, at recent meetings of the Cardiff and Swansea Hospitals. The position of the latter is considered in our Notes this week. At the monthly meeting of the Board of Management of the King Edward VII.'s Hospital, Cardiff, the discussion arose from the issue of the circular letter, approved for publication at the previous meeting and quoted at some length in The Hospital of the 24th ult. It will be remembered that this was an absolutely non-controversial statement, pointing out in unmistakable terms that the work of the hospital would not be superseded by the provisions of the Act, and that the necessity for generous support of the institution was probably greater than at any previous period of its history. It now transpired that the employees of forty-four firms had written to the hospital that they would have difficulty in continuing their subscriptions in consequence of the demands made upon them by the Insurance Act, and the Chairman, Colonel Bruce Vaughan, made a powerful appeal for continued and even more generous support, emphasising the fact that at least an additional £3,000 a year is required before they could venture to open the remaining fifty beds of the new wing. The representatives of the working men present at the meeting gave Colonel Vaughan their assurance of continued support, and the discussion ended in a happy suggestion from Mr. William Jones, the representative of the important Melingriffith Works, near Cardiff, that the realisation of the Board's intention to establish a Workmen's Hospital Society, on the lines so successfully adopted at Leicester, should not longer be delayed.

Following on this, last Saturday fifteen members of the Executive of the Cardiff, Penarth and Barry Coal-trimmers' Union, with the Secretary, Mr. Samuel Fisher, paid a visit to King Edward VII.'s Hospital, Cardiff, and under the guidance of Colonel Bruce Vaughan, the Chairman of the House Committee, made a thorough inspection of the various wards and many special departments of the institution. Before leaving, Mr. Clatworthy, the President of the Union, expressed to Colonel Vaughan their warm appreciation of its equipment and of the good work that is being done, and at a subsequent meeting of the Executive Committee of the Union, held on the same evening, the following resolution—proposed by Mr. T. E. Russell, and seconded by Mr. J. H. Gibby—was carried unanimously: "That the Executive Committee having had the privilege of inspecting the King Edward VII.'s Hospital, Cardiff, regrets to find that, notwithstanding there are nearly 900 patients awaiting admission, the new wards are not yet in use, and respectfully urges upon the authorities of the hospital to equip and open them at the earliest possible date in order that the institution might be used to its full capacity. This Executive Committee pledges itself to do all in its power to raise a good share of the necessary funds to meet the extra cost."

It has been remarked locally in what striking contrast is the action of the coaltrimmers to that of the employees of the forty-four firms above referred to.

